# Top-Down Proteomics and the Challenges of True Proteoform Characterization

**DOI:** 10.1021/acs.jproteome.3c00416

**Published:** 2023-11-08

**Authors:** Allen Po, Claire E. Eyers

**Affiliations:** † Centre for Proteome Research, Faculty of Health & Life Sciences, University of Liverpool, Liverpool L69 7ZB, U.K.; ‡ Department of Biochemistry, Cell & Systems Biology, Institute of Systems, Molecular & Integrative Biology, Faculty of Health & Life Sciences, University of Liverpool, Liverpool L69 7ZB, U.K.

**Keywords:** top-down proteomics, post-translational modification, phosphorylation, proteoform

## Abstract

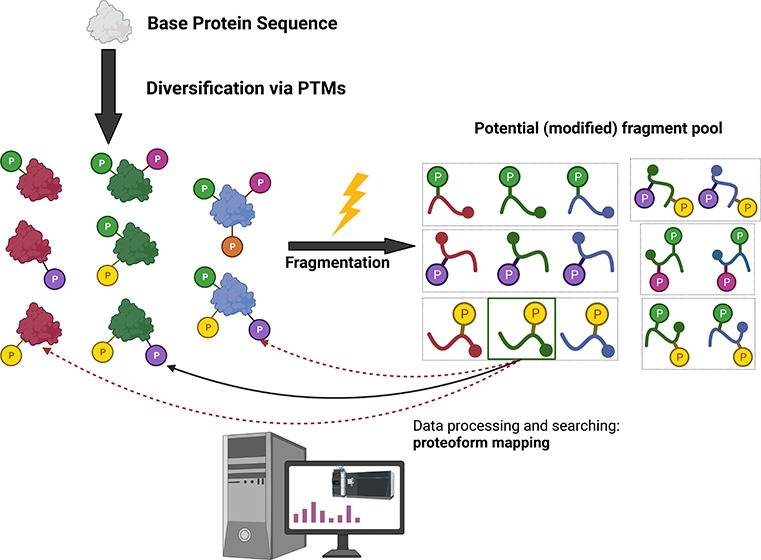

Top-down proteomics (TDP) aims to identify and profile intact protein forms (proteoforms) extracted from biological samples. True proteoform characterization requires that both the base protein sequence be defined and any mass shifts identified, ideally localizing their positions within the protein sequence. Being able to fully elucidate proteoform profiles lends insight into characterizing proteoform-unique roles, and is a crucial aspect of defining protein structure–function relationships and the specific roles of different (combinations of) protein modifications. However, defining and pinpointing protein post-translational modifications (PTMs) on intact proteins remains a challenge. Characterization of (heavily) modified proteins (>∼30 kDa) remains problematic, especially when they exist in a population of similarly modified, or kindred, proteoforms. This issue is compounded as the number of modifications increases, and thus the number of theoretical combinations. Here, we present our perspective on the challenges of analyzing kindred proteoform populations, focusing on annotation of protein modifications on an “average” protein. Furthermore, we discuss the technical requirements to obtain high quality fragmentation spectral data to robustly define site-specific PTMs, and the fact that this is tempered by the time requirements necessary to separate proteoforms in advance of mass spectrometry analysis.

## Introduction

Proteomics, namely analysis of the “entire protein complement expressed by a genome, or by a cell or tissue type” under defined conditions,^1^ is an essential means of exploring physiology and the changes that occur, e.g., during stress, infection, or disease. By collating such information, it is possible to explore protein function and evaluate the effects of targeted intervention of a protein or pathway, for example with small molecule inhibitors. There has been an exponential rise in proteomics outputs since just before the turn of the century that can be attributed to a series of instrument and computational developments that coalesced around that time, including: commercial release of a hybrid quadrupole time-of-flight instrument permitting high resolution tandem mass spectrometry (MS/MS) of selected peptide ions, integration of ultra-high-performance liquid chromatography (UHPLC) (primarily using reversed-phase (RP) chromatographic media) with nanoelectrospray ionization (nESI),^2^ and the increased prevalence of protein databases as well as search algorithms for their interrogation.^3^ However, it is well understood that both cellular and acellular environments can be incredibly complex, and the challenges associated with proteome analysis increase substantially with the size of the analyte.

**Figure 1:**
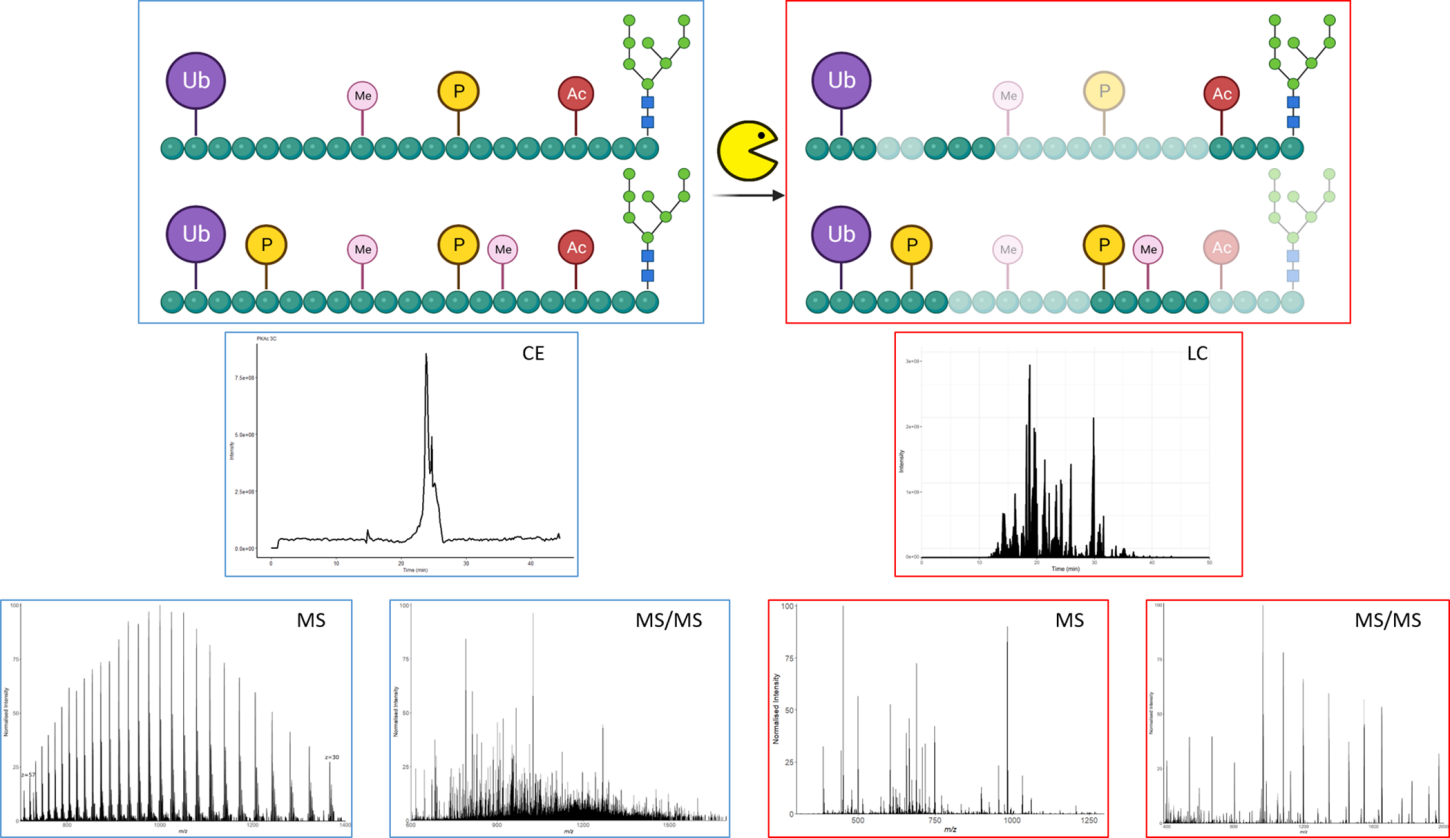
Top-down (left, blue) vs bottom-up (right, red) proteomics: Differences in spectral characteristics and complexity. Top-down investigations allow for the analysis of intact protein sequences and any mass shifts in the form of PTMs, truncations, etc. Bottom-up analyses typically provide more detail on the localization of mass shifts (modifications, SNPs), as well as being comparatively simpler in terms of analytical and computational complexity, but can miss regions of the protein that may be modified. Potential sites and type of modifications are represented (Ub, ubiquitin; Me, methylation; P, phosphorylation; Ac, acetylation); CE, capillary electrophoresis; LC, liquid chromatography. Note that some regions of the protein are not detected following digestion in a bottom up pipeline.

To overcome the analytical issues associated with intact protein characterization, the vast majority of proteomics investigations routinely employ a peptide-based or “bottom-up” strategy, which while incredibly useful, is limited in terms of understanding the true nature of functional proteins (Figure 1). Typically, peptides are generated by proteolysis with enzymes of defined specificity (often trypsin) prior to LC-MS/MS analysis. While proteolysis increases the complexity of the sample being analyzed, this can be overcome using, e.g., chromatographic strategies, and there are substantive benefits from a mass spectrometry perspective that have resulted in this being the primary method of choice for proteomics investigations. However, the downside of peptide-based analysis is that protein sequence coverage is generally incomplete, and there is an inability to reconstruct information pertaining to modification or genetic variation in the context of the whole protein (Figure 1).

Top-down proteomics (TDP) is the study of intact proteins and their proteoforms,^4,5^ a term that describes the complexity of different protein forms that can arise from a single gene, introduced by truncations, post-translational modifications (PTMs), alternative splicing and/or genetic variation. Intact protein analysis can, in theory, define these proteoforms, providing a holistic view of a proteome (Figure 1). However, such analyses are far from trivial. From the ∼20,000 human genes, the number of proteoforms is estimated to be anywhere between 1 million and 6 million different species, considering the combinatorial diversity of different isoforms, single nucleotide polymorphisms (SNPs), and PTMs (covalent modifications, truncations, and chemical modification of amino acid side chains). While these are unlikely to all coexist, there is no doubt that proteoform heterogeneity is extensive, with hundreds of thousands of proteoforms being present in a cell at the same time, each having defined, and possibly unique, biological roles, or similar roles under different conditions.^6−8^

Much progress has been made in the field of top-down proteomics over the last ∼20 years, although there remain numerous challenges particularly for the robust characterization of proteoforms larger than ∼30 kDa and specifically, in the discrimination and localization of PTMs. To further add to some of the clinical work that has already been done,^9−14^ and to truly understand the physiological and disease-specific roles of distinct proteoforms, such information is essential.

In this article, we discuss the multiplexed proteoform challenge: the analytical and computational issues that remain to be overcome to allow true proteoform characterization where both the types and sites of modification can be defined. Specifically, we highlight the challenges associated with characterizing kindred proteoforms that may contain an abundance of potential “action sites”, as they exist in a complex and heterogeneous protein population.

## Benefits and Limitations of Peptide-Based Proteomics

Trypsin was first used to facilitate the analysis of proteins by mass spectrometry in 1970,^15^ and has become the cornerstone of MS-based protein analysis. Tryptic peptides are generally of an ideal size and composition for separation by C_18_ RP chromatography. Following (n)ESI, they also typically yield peptide ions over a limited range of charge states (typically 2+ or 3+) in a relatively compact *m*/*z* window (*m*/*z* ∼ 500–1800) that readily undergo fragmentation in a defined manner.^16^ While not the only method of generating peptides for LC-MS analysis, tryptic proteolysis has undoubtedly become the method of choice, with other proteases or chemical-induced cleavage being used to overcome limitations in protein sequence coverage of tryptolysis products.^8,17−19^

Peptide ion fragmentation is generally very efficient, with collision-induced dissociation (CID), or higher energy collision-induced dissociation (HCD), generating almost complete product ion series enabling unambiguous peptide identification. Ambiguity can arise during automated analysis of highly complex mixtures when peptide ion abundance is low, for chimeric spectra, or for peptides that contain labile, large or complex covalent modifications, e.g., phosphate, glycans, ubiquitin. However, the ability to localize sites of PTM^20−27^ is perhaps one of the greatest advantages of peptide-based proteomics. PTMs play critical roles in diversifying the functions of expressed gene products, and can be regulated rapidly and often reversibly, in a context-specific manner. Consequently, quantitatively defining the type, site and dynamics of protein modification is seen by many as the holy grail of proteomics.

There is no doubt that PTM characterization, even at the peptide level, is more problematic than defining the presence of a specific expressed gene product. As well as being of lower abundance (modifications are seldom stoichiometric), the functional group itself may influence ESI efficiency and thus the relative abundance of modified peptide ions. Coupled with the fact that proton-driven collisional fragmentation can result in altered fragmentation pathways for certain types of covalent modification, tandem mass spectra of modified peptides are often of lower quality. The lability of many covalent modifications means that CID often induces neutral loss of the covalent modification at the expense of peptide backbone fragmentation, which consequently reduces confidence and identification scores following automated searching.^28,29^ That being said, there are robust workflows for the high confidence identification (and quantification) of modified peptides, both using CID/HCD and complementary fragmentation strategies such as electron-transfer dissociation (ETD), alone or in combination (EThcD, ETciD).^24,30−32^ However, as the number of potential sites of modification, or “action sites”, on a peptide increases, the greater the need for complete fragment ion series to define the site of modification on the peptide backbone. This is particularly important when potential action sites are in close proximity as there is a need to generate specific site-localizing ions to enable discrimination. Many computational algorithms now specifically consider the ability to localize PTMs thus providing a measure of confidence as to site assignment, and numerous groups including our own have benchmarked these tools as a function of fragmentation regime.^24,27,33,34^

One of the main problems associated with bottom-up proteomics is protein inference, given that it is the proteolytic peptide, not the protein, which has actually been identified. Many peptides are redundant in terms of the specific protein family member from which they might derive and will match to one or more potential proteins or protein isoforms within a database. Protein, rather than protein group or family “identification”, thus relies on the detection of unique peptides, which may or may not be amenable to LC-MS/MS. As we further consider modified peptides, and the common observation that multiple modified peptides often derive from the same protein family, there is no way to reconfigure the puzzle of their origin. Crucial information regarding PTM interplay and coexistence on a single protein entity is lost within the sea of peptides generated.

The incomplete protein sequence coverage that results following analysis of proteolytic product (Figure 1), combined with the inability to determine the proteoform origin of modified peptides means that bottom-up proteomics cannot to be used to understand proteoform complexity. However, peptide-based investigations that start to define protein and PTM “space” of a biological sample of interest can be incredibly useful to inform top-down database searching, limiting the number and location of potential action sites to those that are known or have been previously observed.

## Challenges of Top-Down Proteomics

“Top-down” characterization of intact proteins was first described in 1999 by Neil Kelleher, Fred McLafferty, and colleagues, where they used Fourier transform (FT) MS for sequence determination of the 29 kDa protein carbonic anhydrase B.^35^ Since then, there has been a steady increase in the number of top-down proteomics investigations, including several seminal projects.^13,36,37^ However, TDP has not taken off in the same way as bottom-up analysis despite its obvious benefits. To help drive work in this area, Neil Kelleher and colleagues launched the Consortium for Top-Down Proteomics^36,38^ in 2012, bringing the community together to promote collaboration and resource sharing. A number of reviews have been published detailing the history of TDP, practicalities in terms of implementation, and potential future directions in the field,^39−47^ some aspects of which are discussed below.

Intact analysis comes with its own specific challenges, most of which relate to the need for high quality, high resolution fragmentation spectra, and the interpretation of those data which are far more complex than for peptide-based investigations. While the complexity of top-down proteomics samples is much reduced compared with bottom-up, the analytical complexity is greater due to broader charge state distributions and the substantial increase in the number of product ions generated from isolated species.^48^ Distribution of ion current across a higher number of charge states, and the fact that ESI efficiency is generally lower for proteins compared with peptides, means that this type of experiment is much less sensitive. Bottom-up analysis also benefits from redundant protein group/family derived peptides which provide a signal boost for identification of gene expression products that is not permissible with protein level studies. A number of strategies have been implemented in an attempt to boost the analysis of low abundant proteins for TDP, including functionalized nanoparticles^49^ (NPs) and nanodroplet sample processing to improve recovery of proteins for downstream analysis.^50^ Functionalized NPs have been developed specifically for phosphoproteins,^51^ which possibly defeats the idea of investigating the complete proteoform landscape but may be useful if solely interested in phosphoproteoforms. Other NPs have been developed for specific classes of protein, such as the work described by Ying Ge and colleagues who used peptide-functionalized NPs to investigate cardiac troponin from human serum.^52^

**Figure 2:**
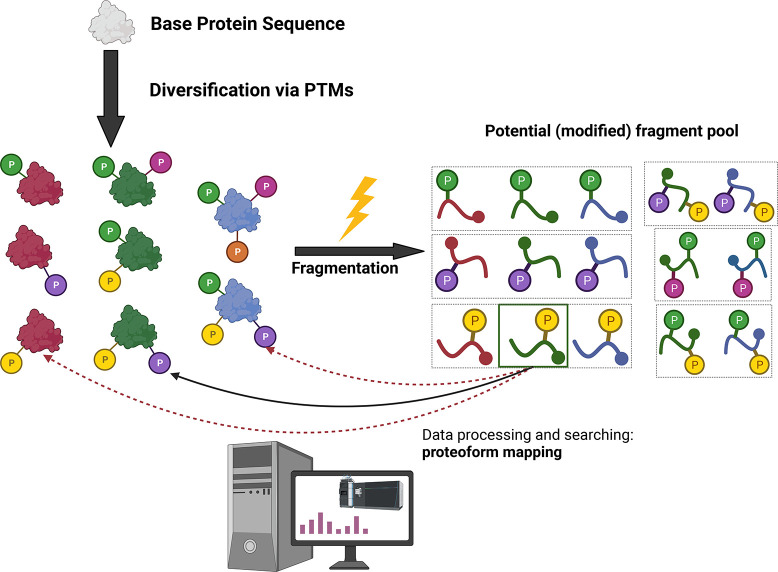
Challenges of kindred proteoform analysis. Kindred proteoforms (proteins as red, green, or blue) that have the same base sequence and the same modification type (P, phosphorylation) but in different numbers and at different sites (represented by a different color P) add to analytical difficulty. Identical fragment ions (boxed) can be produced from different kindred forms. A specific product ion could then be matched following data processing to different, possibly incorrect, proteoform species (as exemplified by the product ion in the green box) which is exacerbated in lower resolution data.

Intact protein analysis is ideally suited to PTM profiling, that being elucidation of the number and types of modifications. Using MS2 data to define the base protein sequence, the difference between the theoretical and experimentally determined mass (at MS1 level) can be used to define the proteoform landscape, indicating the number and type of modifications, bolstered by data as reported in databases such as UniProt. Many current studies consider this sufficient for proteoform identification, yet to understand the biological complexity and specific functions of different proteoforms, we need to go one step further—we need to determine not only the PTM profile, but also define the sites of modifications on the protein backbone. Only by so doing can we understand the interplay and hierarchy of different PTMs, important in the context of exploring their regulatory effects. Recognizing that TDP challenges also include the need for solutions that address issues with protein solubility and enrichment of low abundance proteoforms (which have been reviewed elsewhere),^53,54^ we focus here on proteoform complexity, and specifically the ability to differentiate and define kindred proteoforms (Figure 2).

## Proteoform Populations and Kindred Proteoforms

The ideal goal of a TDP experiment is the unambiguous characterization of all proteoforms of relevance to understand a given biological system in an untargeted manner, be that changes in the proteoforms of a specific protein family as has been undertaken extensively for histones^55,56^ (with kindred proteoforms being derived from a single gene), or more broadly (proteoform populations) as relevant to defining drivers or biomarkers of disease.^14^ Rather than making a challenging system more complicated, we will focus here on the issues that arise when attempting to define all proteoforms of a given family as could be achieved, e.g., following immunoprecipitation.

Histones have long been the “poster child” of the TDP movement as their relatively small size means that analytical complications are reduced compared with an average protein (∼38 kDa in humans). Histone PTM is a well-established means of epigenetic regulation, influencing chromatin structure and the recruitment of chromatin binding proteins.^57−59^ Consequently, there has been a real biological incentive to focus on defining variation in the “histone code” and how these relate to gene expression; abnormal histone PTM fingerprints are markers of numerous diseases including cancer^58,60−62^ and autoimmune diseases,^63^ as well as being regulated as a function of, e.g., age.^64^ As an example of the complexity identified even in these relatively small proteins, Holt et al. identified over 600 potential proteoforms of the 11 kDa histone H4 protein, using a bespoke pipeline to quantify around 200 biological proteoforms as a function of treatment of a triple negative breast cancer cell line.^55^ However, as protein size increases, the ability to confidently define kindred proteoforms decreases due to issues associated with proteoform separation and the ability to generate sufficient site-localizing product ions.

**Figure 3:**
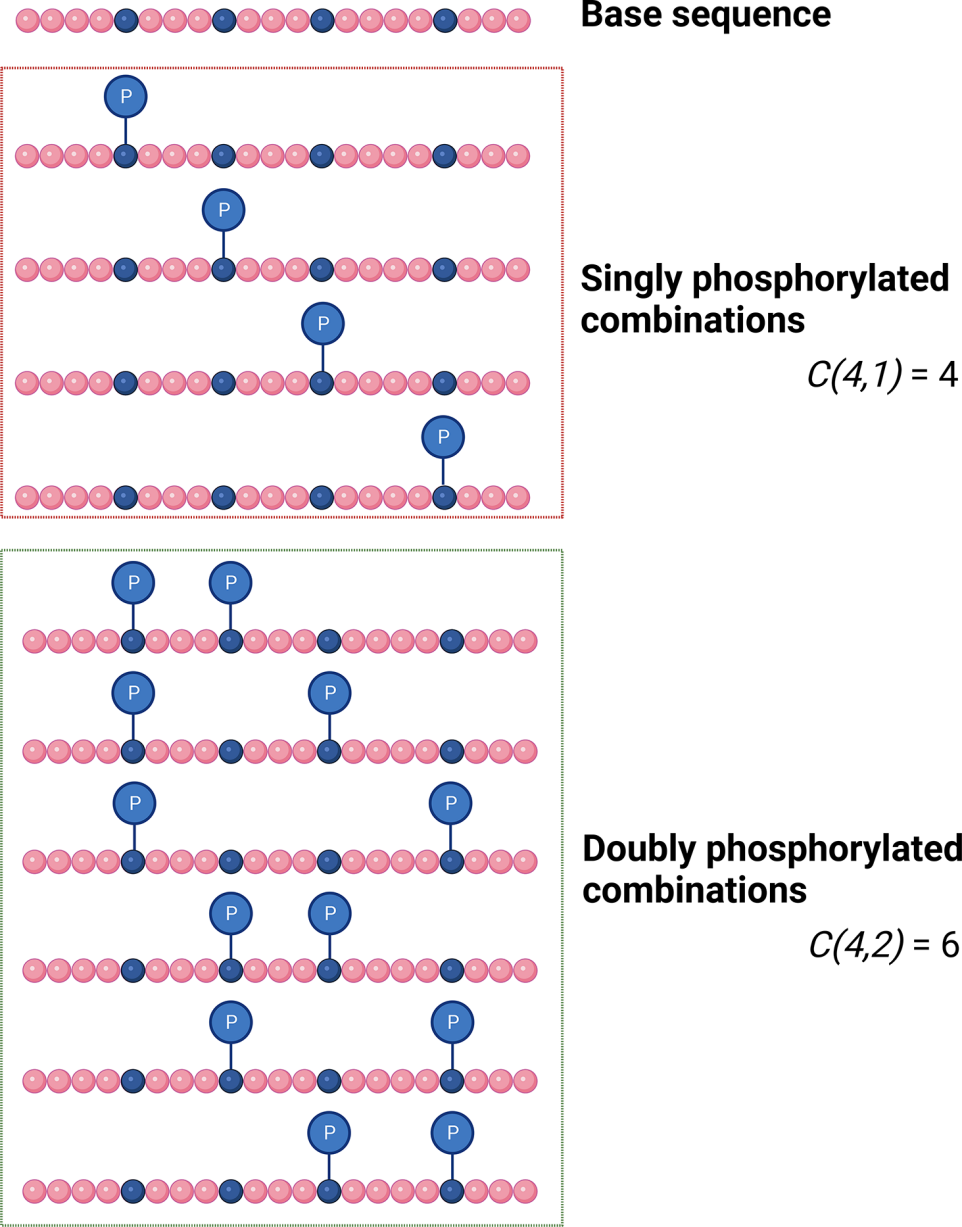
Combinations of theoretical isobaric proteoforms. The number of potential proteoforms for a given base protein sequence of a defined precursor mass (*C*) is dependent on the number of potential action sites (in this case phosphorylation; *n* = 4) and the number of observed events (*r* = either 1 (top) or 2 (bottom)) as defined by the difference in mass between the base protein (theoretical) and the experimentally observed proteoform.

Consider a ∼45 kDa protein such as the catalytic subunit of the archetypal protein kinase, protein kinase A (PKAc): it has 45 potential phosphorylation sites (counting Ser/Thr/Tyr residues); there is strong evidence for phosphorylation of 11 sites, and additional evidence that a further 7 may be modified.^65^ Intact analysis suggests that the protein exists as a population of proteoforms containing between 5 and 9 phosphorylation events, which may themselves be isobaric (i.e., the same number of phosphate groups located at different sites).^65^ The number of potential combinations for a given precursor population at a defined *m*/*z* can be calculated as
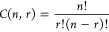
where *n* is the number of potential action sites and *r* is the number of observed events (Figure 3).

Thus, considering all potential sites of phosphorylation, there are 1.2 × 10^6^ possible proteoforms for the 5 phosphate-containing precursor, expanding to 8.9 × 10^8^ theoretical proteoform combinations with 9 phosphate groups. If we constrain this space based on peptide observational data, then we still have 462 potential combinations for the 5 phosphate-containing species. Realistically, not all these combinations are likely, and indeed our understanding of proteins such as PKAc would suggest a hierarchy of phosphorylation with some sites being preferentially incorporated before others.^65,66^ While this example only considers phosphorylation (Figure 3), other co-occurring PTMs are common: for example, PKAc is known to be additionally modified by deamidation, myristoylation, ubiquitination and possibly glycosylation, with numerous additional disease-related SNPs. However, the challenge of unraveling this type of information for kindred proteoforms remains an important element to drive protein and proteoform specific biological understanding.

## Proteoform Separation—The Issue of Chimeric Spectra

**Figure 4:**
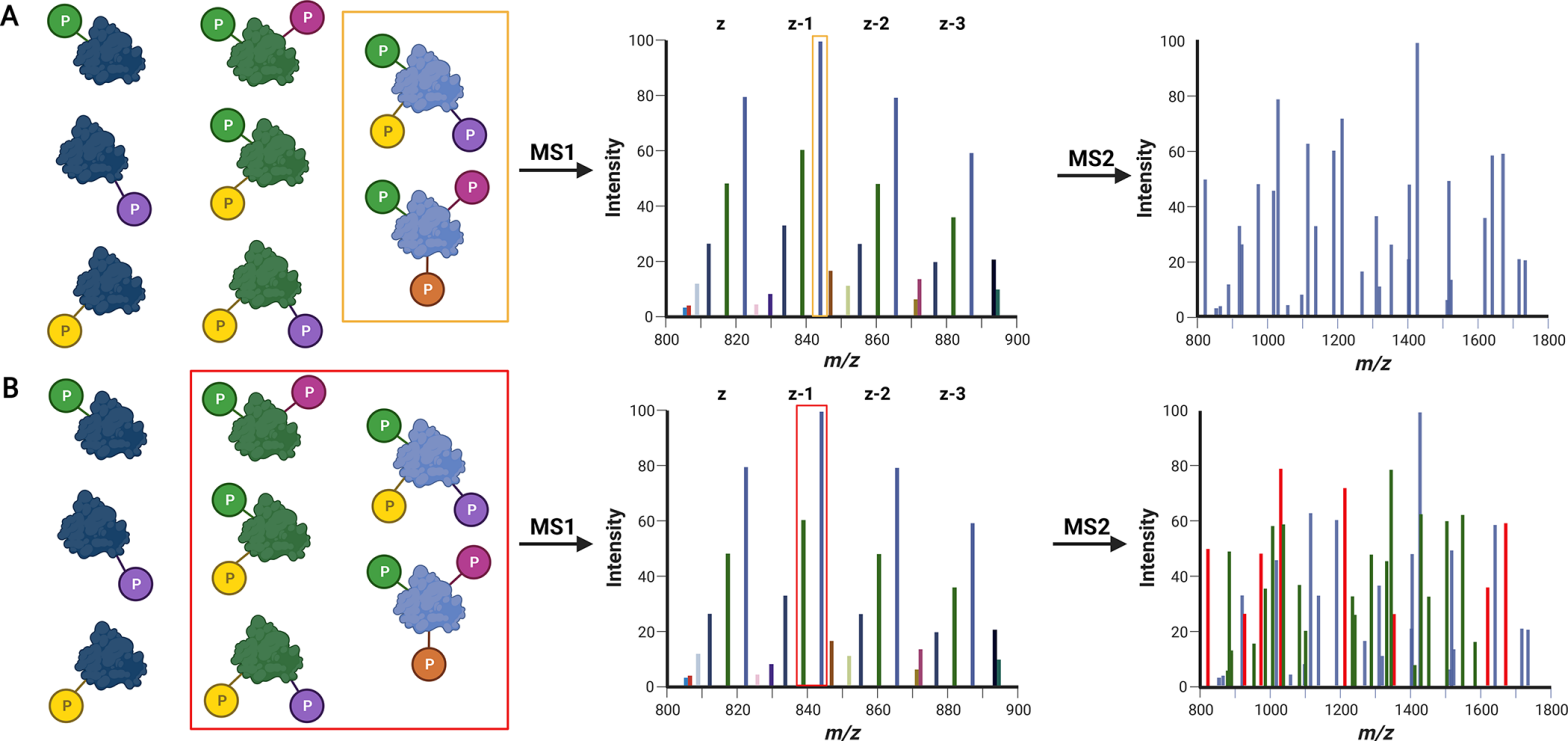
Effect of isolation width on the generation of chimeric MS spectra. Kindred species (left) that contain the same base sequence and the same types of modification (P, phosphorylation) but in different quantities increase analytical complexity. (A) A narrow isolation window for MS2 prevents coisolation of similar species, thus generating isoform-specific MS2 spectra, but does not overcome the issue of isobaric proteoforms. (B) Too wide an isolation window can lead to ambiguity for precursor assignment as kindred species yield identical (and similar) fragment ions. Red ions in the MS2 spectrum equate to fragment ions that could derive from a number of kindred proteoform.

**Figure 5:**
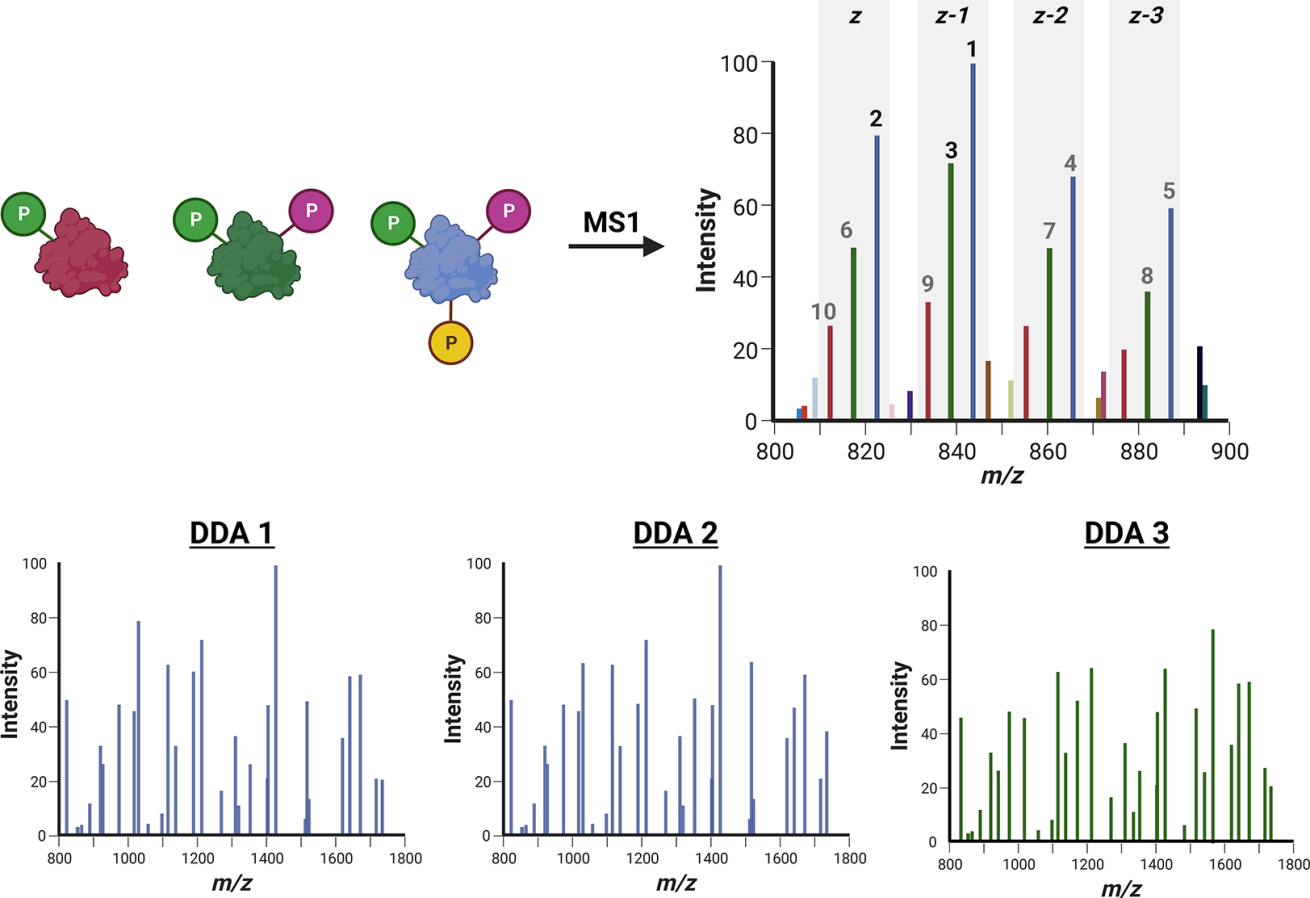
Proteoform selection for MS2 with data-dependent acquisition (DDA). Where precursor signal intensity is used to select ions for fragmentation, the presence of multiple charge states (*z*, *z* – 1, *z* – 2, *z* – 3) of a single proteoform (represented here as either red, blue, and green) can result in isolation and fragmentation of different charge states of the same species, reducing diversity of information and the number of proteoforms that can be identified. In this example, representative of a Top10 DDA experiment of kindred proteoforms, two charge states (*z* – 1; *z*) of the 3 phosphate-containing species (blue) are selected before the most intense charge state (*z*) of the doubly phosphorylated proteoform (green). The singly phosphorylated proteoform is the 9th most abundant ion in this example and thus may not be selected depending on the duty cycle. MS/MS spectra of different charge states of the same proteoform (e.g., DDA1 and DDA2) may differ marginally due to slight differences in protonation density and gas-phase conformation.

To uncover proteoform diversity, it is important to be able to generate MS/MS spectra free from near isobaric contamination (as far as is feasible). Specific proteoforms must be isolated to minimize the generation of chimeric spectra and thus facilitate the assignment of proteoform-specific product ions (Figure 4). Chimeric spectra, or tandem mass spectra which are sparse in product ions (discussed further below), may contribute to defining proteoform landscape, but not the identification of specific proteoforms. Chimeric spectra are also more likely to result in potential false positive matches or ambiguous site assignment following data analysis. Separation of different protein species in advance of MS analysis also serves to capture lower abundant species which may not otherwise be isolated and thus selected for fragmentation in a data-dependent acquisition (DDA) style experiment (Figure 5).

The key to evaluating kindred proteoforms is the ability to separate them from each other in advance of MS2 analysis. Gel-eluted liquid fraction entrapment electrophoresis (GELFrEE) has proven extremely useful for simplifying complex protein extracts from cells and tissues for TDP analysis.^67−71^ However, by itself it does not improve the ability to identify related proteoforms, separating as it does on the basis of mass at low resolution.^72^ Coupled with a customized solution isoelectric focusing (sIEF) device and online RP HPLC system, GELFrEE has proven efficacious for improving proteoform identification including from clinical samples.^73,74^

In our scenario where we aim to define differentially modified proteoforms of a given family, isolation for MS/MS analysis is best accomplished by front-end separation and quadrupole isolation within the mass spectrometer. As for bottom-up proteomics, RP UPLC remains the method of choice for online separation because of its compatibility with (n)ESI. There have been substantive improvements in the separation of proteins for TDP by RP chromatography over recent years due to the development of protein-specific UPLC (and HPLC) media, with monolithic columns functionalized with short (C_4_–C_8_) alkyl chains becoming popular.^75−78^ As important as RP chromatography has and will continue to be for TDP, separation based on hydrophobicity inherently limits the ability to separate large proteoforms that may differ by small modifications, e.g., oxidation, acetylation, phosphorylation. In this regard, (online) capillary electrophoresis (CE) has shown some promise, proving to be an orthogonal separation strategy to RP chromatography in its ability to separate analytes for identification.^37^ Capillary zone electrophoresis (CZE) separates species according to their charge-to-size (frictional coefficient) ratio which can be tailored depending on the background electrolyte and the capillary coating. CZE also allows for highly efficient separations with over 1,000,000 theoretical plates being possible for intact proteins due to the low diffusion coefficient of large molecules.^79^ As such, it offers the potential for separation of kindred proteoforms that are largely inaccessible by RP, particularly those that alter the charge on a side chain as seen with, e.g., deamidation or phosphorylation, or some SNPs.^80−85^ Operating at much lower flow rates (10s of nL/min) and with the ability to apply sample stacking as an online preconcentration step,^79^ CE improves the limit of detection, which is particularly pertinent for identification of low abundant proteoforms.^82^ Indeed, a recent study by Johnson et al. reported the identification of up to 50 proteoforms from a single human HeLa cell, demonstrating applicability of CE-MS/MS even for extremely low sample quantities.^86^

Multidimensional (2D/3D) separations are also possible, and combining orthogonal separation methods increase the chance of identifying low abundance species. However, this increase in proteoform coverage typically comes at the expense of instrument time, requiring multiple acquisition runs. These types of multidimensional workflow can also lead to sample dilution or the need for additional cleanup steps that can contribute to sample loss. Ion exchange chromatography (IEC) and hydrophilic interaction chromatography (HILIC) have been employed for large scale proteoform identification, being used as part of 2D/3D workflows prior to online RP LC,^54,87−89^ and occasionally as online separation systems. However, these generally lack the benefits (increased sensitivity and resolution capabilities) of CZE for separation of kindred proteoforms.

Even the best strategy for proteoform separation is currently unable to baseline resolve closely related proteoforms, with separation capacity decreasing for larger proteins that are minimally modified. Consequently, the parameters for precursor isolation (instrument resolution and isolation width) are critical to generating high-quality tandem MS data for a single proteoform (Figure 4). There is also an inherent “salary cap” in terms of experimental resolution and cycle time capabilities. Considering the Tribrid Orbitrap platforms, doubling the MS resolution from 120k to 240k will double the scan time. Thus, overall cycle time must be constrained for studies that require online proteoform separation; MS2 data quality will be compromised for cycle times that are longer than half-height peak width of the separation given that the number of ions available for isolation and fragmentation will be reduced. There is therefore a balance between resolution needs, the ability to capture as many low abundance species as possible, and the quality of the MS2 spectra that can be generated.

Another confounding factor is isobaric proteoforms—members of a proteoform family that contain the same number and type of PTMs, but where covalent modifications are localized at different residues. These will not separated by mass or charge and consequently will generate chimeric tandem mass spectra upon isolation. This phenomenon is well understood in the context of peptide-based phosphoproteomics studies resulting in coelution of isobaric phosphopeptides that generate ambiguous site localization scores.^24,90^ Ideally, such isobaric proteoforms should be separated in advance of MS analysis to minimize the generation of chimeric spectra, deconvolution of which is heavily reliant on computational software.

Thinking about the 462 potential combinations for the 5 phosphate-containing species of PKA_C_, even assuming that there are only 10 such proteoforms, their identification relies on the ability to generate and identify site localizing ions using current TDP algorithms. One beacon of light for the analysis of isobaric proteoforms will be conformational-based separation. Working on the assumption that these isobaric proteoforms have specific biological functions then it stands to reason that there will be some difference in their conformation. Ion mobility spectrometry (IMS) can be operated in-line on the time scale of an (LC/CE)-MS experiment and has the potential to separate either precursor or product ions based on differences in their structure.^91,92^ While there are a small number of studies describing the application of IMS for TDP,^91,93^ this is an area that has great potential for the field, but its real utility will depend on the ability to conformationally resolve isobaric kindred proteoforms. Data generated by our group and others also suggests that CE using a background electrolyte at near physiological pH may be suitable for the conformational separation of such proteoforms, and this remains to be explored.^94^

## Proteoform Isolation and Fragmentation

In DDA approaches, ions are selected for fragmentation based on relative ion intensity in an MS1 survey scan. A common problem, particularly in the analysis of kindred proteoforms, is repeat selection of different charge states of the same species (Figure 5). Repeat sampling can be beneficial, increasing confidence in proteoform identification. Different charge states of the same proteoform can also generate different product ions, with ions of greater charge state (but generally of lower relative abundance) typically yielding more product ions as precursors are less structured. However, repeated selection of the same proteoform will decrease the likelihood of identifying kindred species. A number of strategies have and can be used to overcome this: (1) decreasing the observed number of charge states for a given species through the use of supercharging reagents such as sulfolane or propylene carbonate;^95−98^ (2) reducing the *m*/*z* range for MS1 acquisition such that a single charge state is selected for all proteoforms (Figure 4); (3) generation of an inclusion list for subsequent isolation and fragmentation based on the optimal charge state for each proteoform—the most intense, or those predicted to yield the most informative product ions. Supercharging reagents can be advantageous in that a survey scan is not needed to define MS acquisition parameters (as required for options 2 and 3). However, inclusion of such additives either needs to be compatible with any online separation, or added post separation (e.g., through a T-junction) prior to nESI. Further development and implementation of strategies that simplify the number of charge states at the MS1 level, or are capable of isolating (and subsequently fragmenting) multiple charge states of the same species (e.g., through multi-ion quadrupole isolation, or multinotch isolation in an ion trapping device) thus have great potential for TDP and the analysis of kindred proteoforms.

The next step for consideration is precursor ion isolation: if preanalytical protein separation strategies are sufficient, it is technically feasible to isolate ions from a broad *m*/*z* window, selecting multiple charge states of the same precursor and any kindred proteoforms within the window; this can undoubtedly boost signal and MS2 spectral quality. However, the ability to differentiate kindred species and correlate site determining product ions with the relevant precursor ion from a wide isolation window becomes a substantive computational challenge (Figure 2). Identification of proteoforms of low relative abundance will be particularly problematic. Consequently, optimal identification of kindred proteoforms will likely best be achieved using narrower isolation windows specific for the proteoform in question, accumulating microscans, with or without multicharge state selection, to improve MS2 quality for identification purposes.

Characterization of analyte structure using tandem mass spectrometry requires consideration of both the chemistry driving the fragmentation process, and its efficiency. To define the site(s) of modification and differentiate kindred proteoforms, efficient fragmentation with *m*/*z* measurement at sufficient resolution to determine product ion charge state is essential. For proteins, fragmentation efficiency is typically greater toward the termini of the sequence, with coverage toward the middle often severely lacking. This disparity in fragmentation efficiency becomes more pronounced for larger proteins and is thought to arise due to residual higher order structure, even under denaturing conditions, which limits accessibility. Even when larger fragments are generated, deconvolution of highly charged product ions that are present in dense regions of MS/MS spectra can be problematic, and current search algorithms are severely limited in their ability to consider internal product ions. While inefficient fragmentation is less of a problem for defining proteoform landscape, it becomes a substantive issue for proteoform characterization in its true sense, as fewer product ions are generated that may permit unambiguous PTM site localization. Inefficient fragmentation is particularly problematic for differentiating proteoforms known to contain multiple centrally localized PTMs. Sequence coverage, and thus the ability to generate site determining product ions, can be improved by application of different fragmentation regimes which use different pathways for dissociation in separate experiments, and then combining the fragment maps. Tools such as the R package *topdownr*(99) are now available to integrate these data and this feature should be routine in all new TDP software. High energy collision-induced dissociation (beam-type CID, or HCD as termed in the Orbitrap series of instruments) is the most commonly applied fragmentation strategy for TDP and relies on collision of protein ions with inert gas atoms/molecules to induce fragmentation, primarily directed toward regions of lower gas-phase basicity which can be protonated. While CID is notorious in the bottom-up proteomics field for being problematic for PTM site assignment due to neutral loss of labile covalent PTMs from the protein backbone, this effect is generally less pronounced in proteins due to redistribution of the collision energy across the larger analyte.^100−102^ Electron capture dissociation (ECD) and particularly ETD are generally more useful for characterizing multiply charged post-translationally modified polypeptides and provide more uniform distribution of fragment ions. However, ETD (and EThcD/ETciD) can suffer from being slow and inefficient.^103,104^ Application of ultraviolet photodissociation (UVPD) is also starting to prove useful for enhanced sequence coverage, or more typically the generation of complementary ions, over those observed with collision-induced or electron-mediated approaches.^105^ Assignment of (typically ETD generated) product ions can also be facilitated by “proton transfer” reaction (proton transfer/charge reduction, PTR) reducing product ion charge state which serves to reduce spectral complexity and thus the ease of identification.^106,107^

Ultimately, the MS acquisition workflow needs to be optimized for the protein family and the system under investigation; acquisition parameters (the number of microscans, resolution, isolation width, etc.) and fragmentation regimes need to be defined depending on the number of kindred proteoforms present and time-constraints of any online separation. Optimization of each node of the workflow presents its own specific challenges, and cannot generally be considered in isolation given their interdependence in terms of generating spectra of sufficient quality. A Design of Experiments (DoE) type approach should thus be considered,^108^ although this is often problematic to perform in its truest sense unless there is an ability to control both data acquisition and analysis in real-time. Ultimately, acquisition parameters should be set based on time allowance and desired outcomes of the study. Scheffler et al.^109^ present an interesting example of TDP workflow optimization for simple mixtures (recombinant proteins and antibodies) using Thermo Scientific Orbitrap platforms (a Q Exactive HF hybrid quadrupole-Orbitrap mass spectrometer and an Orbitrap Fusion Lumos Tribrid instrument). This article along with others published by Neil Kelleher and colleagues, e.g., ref (110) and resources provided by the Consortium for Top-Down Proteomics (https://www.topdownproteomics.org/resources/methods/), provide a useful starting point for drafting analysis (and sample preparation) strategies.

## Data Interrogation

Search algorithms and software for data analysis is a critically important facet of all proteomics pipelines, and TDP is no exception. The past few years has seen development of a variety of different software packages for top-down data interrogation: the Thermo Scientific licensed ProSightPD built on ProSight Lite, was developed by Fellers et al. for simple comparison of MS data against a single candidate sequence.^111,112^ As TDP has become more popular, so has the release of user-friendly, open source tools such as the MASH Suite package,^113−116^ and TopPIC Suite,^117−119^ which consider spectral deconvolution and data processing through to database searching and informative data representation tools. Most freely available packages are also compatible with the different types of input data generated from the range of available TDP capable instrumentation. A comprehensive and very informative comparison of some of these software tools by Tabb et al.^120^ promotes the use of multiple different search engines for data, as has previously been suggested for high throughput bottom-up proteomics studies (although is now rarely implemented).^121^

A substantive challenge in the computational analysis of top-down data, particularly for kindred proteoforms, is understanding and defining PTM site ambiguity. Oftentimes, PTM sites will be “localized” by software based on little to no site-determining product ions. Understanding the difference between confident site assignments, as opposed to defining a region within a protein that contains an unlocalized PTM is important for biological interpretation. Yet, few software tools readily highlight this difference. The generation of similar fragmentation patterns from kindred proteoforms can also challenge software tools. Co-isolation of related (nonisobaric) species can mean that product ions are incorrectly assigned to the wrong precursor ion. Narrow isolation windows that minimize precursor ion coisolation combined with enhanced proteoform separation strategies (as discussed above) are thus essential to aid automated data analysis tools and minimize ambiguity in proteoform characterization.

## Outlook

We believe that we are at a cross-roads in TDP: while the field has advanced sufficiently to be able to explore the proteoform diversity of even quite complex samples, there remain substantial challenges in the precise proteoform characterization required to define structure–function relationships. Many aspects of the TDP pipeline need to be considered when optimizing or developing suitable workflows, with data analysis strategies and software being as important as the approaches and instrumentation used for proteoform separation, fragmentation, and analysis. There is substantial scope for development and integration of online strategies for the separation of kindred proteoforms using techniques such as CZE that will facilitate identification and site localization, an added benefit of which will likely be improvements in the ability to characterize low abundance species. Separation in a time dimension would enhance MS2 data quality, minimizing the generation of chimeric spectra, assuming that cycle times are sufficient to generate spectra of sufficient quality. In this regard, one of the biggest constraints is the ability to routinely and accurately determine charge states of product ions derived from large precursors, and the ability to define and assign internal products.

Data processing software that functions to both deconvolute and search data are essential. The ability to readily incorporate PTM information either from open access databases, e.g., UniProt, or sample specific modifications identified from bottom-up or middle-down analyses is also important to constrain search space. Such software exists, but requires additional concerted development alongside advances in experimental workflows and instrumentation to precisely define PTMs on related proteoforms. In the case of kindred proteoforms, algorithms should also be trained to limit potential false positive matches bearing in mind that substantive portions of their theoretical and experimental fragment lists are often identical. Finally, defining ambiguity in PTM localization is just as crucial as the ability to localize these modifications, allowing experimentalists to explore functional relevance with the required level of confidence in the data.

Over and above proteoform identification, there is also a need to consider proteoform-level quantification. Current evidence suggests that quantification at the intact protein level may be less problematic than peptide-based quantification, as normalized signal intensities for each charge state of a proteoform can be used to determine their relative abundance in a single experiment.^122^ However, MS1-based quantification does not address the issue of positional isomers which will likely necessitate the use of (combinations of) site localizing ions. Future developments in this area should bear in mind the need for proteoform quantification, including for isobaric species, both within a single analytical run and across experiments.

Perhaps the most pivotal question that true top-down characterization is in a position to address is whether unique modification profiles contribute to unique functionality, as has been observed for histone PTM fingerprints. If developments in TDP workflows can be achieved as outlined above, we believe that there will be a step change in the utility of TDP, getting to the heart of defining protein function.

## Abbreviations

CE: capillary electrophoresis
CZE: capillary zone electrophoresis
CID: collision-induced dissociation
DDA: data-dependent acquisition
DoE: design of experiments
ECD: electron capture dissociation
ETD: electron-transfer dissociation
GELFrEE: gel-eluted liquid fraction entrapment electrophoresis
HCD: higher energy collision-induced dissociation
HILIC: hydrophilic interaction chromatography
IEC: ion exchange chromatography
IMS: ion mobility spectrometry
MS/MS: tandem mass spectrometry
(n)ESI: (nano)electrospray ionization
NP: nanoparticles
PKAc: catalytic subunit of protein kinase A
PTMs: post-translational modifications
PTR: proton transfer/charge reduction
RP: reversed-phase
sIEF: solution isoelectric focusing
SNPs: single nucleotide polymorphisms
TDP: top-down proteomics
UHPLC: ultra-high-performance liquid chromatography
UVPD: ultraviolet photodissociation.
